# Effect of Varying Molecular Weight of Oat β-Glucan Taken just before Eating on Postprandial Glycemic Response in Healthy Humans

**DOI:** 10.3390/nu12082275

**Published:** 2020-07-29

**Authors:** Thomas M. S. Wolever, Outi Mattila, Natalia Rosa-Sibakov, Susan M. Tosh, Alexandra L. Jenkins, Adish Ezatagha, Ruedi Duss, Robert E. Steinert

**Affiliations:** 1 INQUIS Clinical Research, Ltd. (formerly GI Labs), Toronto, ON M5C 2N8, Canada; twolever@inquis.com (T.M.S.W.); alexandrajenkins@inquis.com (A.L.J.); aezatagha@inquis.com (A.E.); 2 VTT Technical Research Centre of Finland Ltd., 1000 Espoo, Finland; Outi.Mattila@vtt.fi (O.M.); Natalia.Rosa-Sibakov@vtt.fi (N.R.-S.); 3 School of Nutrition Sciences, University of Ottawa, Ottawa, ON K1N 6N5, Canada; Susan.Tosh@uottawa.ca; 4 DSM Nutritional Products Ltd., R&D Human Nutrition and Health, 4002 Basel, Switzerland; Ruedi.Duss@dsm.com; 5 Department of Surgery, Division of Visceral and Transplantation Surgery, University Hospital Zürich, 8091 Zürich, Switzerland

**Keywords:** humans, oat β-glucan, acute glycemic response, dietary fiber, preload, carbohydrates

## Abstract

To see if the molecular weight (MW) and viscosity of oat β-glucan (OBG) when taken before eating determine its effect on postprandial glycemic responses (PPRG), healthy overnight-fasted subjects (*n* = 16) were studied on eight separate occasions. Subjects consumed 200 mL water alone (Control) or with 4 g OBG varying in MW and viscosity followed, 2–3 min later, by 113 g white-bread. Blood was taken fasting and at 15, 30, 45, 60, 90, and 120 min after starting to eat. None of the OBG treatments differed significantly from the Control for the a-priori primary endpoint of glucose peak-rise or secondary endpoint of incremental area-under-the-curve (iAUC) over 0–120 min. However, significant differences from the Control were seen for glucose iAUC over 0–45 min and time to peak (TTP) glucose. Lower log(MW) and log(viscosity) were associated with higher iAUC 0–45 (*p* < 0.001) and shorter TTP (*p* < 0.001). We conclude that when 4 g OBG is taken as a preload, reducing MW does not affect glucose peak rise or iAUC0-120, but rather accelerates the rise in blood glucose and reduces the time it takes glucose to reach the peak. However, this is based on post-hoc calculation of iAUC0-45 and TTP and needs to be confirmed in a subsequent study.

## 1. Introduction

Reducing the postprandial glycemic response (PPGR) is generally considered to be a beneficial effect [1,2]. Oats and oat products elicit lower glycemic responses than most other types of ready-to-eat and cooked breakfast cereals when comparing equivalent amounts of available carbohydrate (avCHO) [3,4,5,6,7]. Oatmeal is rich in β-glucan, a highly viscous soluble dietary fiber found predominantly in the endospermic cell wall of oats and barley [8] thought to be responsible, at least in part, for the low glycemic impact of oats. Oat β-glucan (OBG) is thought to reduce PPGR by increasing the viscosity of the contents of the upper gut [8,9], which, in turn, may slow gastric emptying, reduce the rate of starch digestion, and increase the thickness of the so-called unstirred water layer in the small intestine, thus delaying the absorption of carbohydrates [9]. The addition of OBG to test meals results in a dose-dependent reduction in PPGR, an effect which is evident with as little as 0.04 g OBG per gram avCHO in the test meal [10].

Previous studies suggest that taking viscous fibers separate from a test-meal may not reduce the PPGR [11,12]. Thus, in order to reduce PPGR, it may be necessary to mix viscous fibers with the food; however, this alters the taste and texture of foods in a way that may reduce palatability. A potential solution to this problem may be to consume a fiber ‘preload’ which develops viscosity slowly so that it can be consumed in a palatable form just before eating and remains dispersed in the stomach long enough to be able to mix effectively with the main meal, but becomes viscous by the time the stomach starts to empty. To this end, we showed that taking commercial oat bran containing 0.9, 2.6, or 5.3 g OBG mixed in water a few minutes before consuming 50 g avCHO as white bread reduced postprandial glycemic responses in a dose-dependent fashion [13]. Since the different doses of OBG were mixed into a fixed amount of water, the viscosity of the preload increased as the dose of OBG increased. Thus, it is not possible to determine from that experiment whether it was the amount of OBG or the viscosity of the preload which affected the glycemic response. We hypothesized that the extent to which an OBG preload reduces the glycemic response depends on its viscosity. Since the viscosity of solutions containing a fixed amount of OBG can be changed by altering the molecular weight (MW) of the OBG, the purpose of this study was to determine the effect of varying the MW, and hence viscosity, of 4 g OBG preloads on the PPGR elicited by a 51.5 g avCHO portion of white bread.

## 2. Material and Methods

### 2.1. Subjects and Study Design

We conducted an open-label study with a randomized cross-over design at a contract research organization; participant visits occurred between 3 January and 6 February 2018. We recruited 16 healthy subjects (9 males and 7 non-pregnant females) of mixed ethnic background (7 Caucasian and 9 non-Caucasian as follows: 4 East- or South-East Asian, 2 South Asian, 2 Hispanic and 1 mixed Aboriginal/Caucasian), aged (mean ± SD) 41 ± 11 y with body mass index 25.6 ± 2.7 kg/m^2^, and all 16 completed the study according to protocol. Compared to the 7 Caucasian participants, the 9 non-Caucasian participants were younger (mean ± SD, 36.4 ± 10.8 vs. 45.7 ± 8.6 y, *p* = 0.084) and had a lower BMI (23.9 ± 1.6 vs. 27.8 ± 2.1 kg/m^2^, pp = 0.001) (Supplemental Table S1). Twelve (12) participants took no prescription medications or supplements, 3 took daily supplements (1 took 750 mg omega-3 fatty acids, 125 mg calcium, and 125 mg magnesium daily; 1 took 2 capsules of the hair growth supplement Priorin daily; and 1 took 1000 mg omega-3 fatty acids and 1000 IU vitamin D daily), and 1 took a birth control pill. The procedures used were in accordance with the protocol used at INQUIS for determining the GI of foods, which was approved by the Western Institutional Review Board^®^. The work described in this manuscript was carried out in accordance with The Code of Ethics of the World Medical Association (Declaration of Helsinki) for experiments involving human subjects. The protocol used was approved by the Western Institutional Review Board^®^ (WIRB) which meets all requirements of the US Food and Drug Administration (FDA), the Department of Health and Human Services (DHHS), the Canadian Health Protection Branch (HPB), Canadian Institutes for Health Research (CIHR) and the European Community Guidelines. Prior to their participation, all subjects provided written informed consent by signing the approved consent form (WIRB protocol number: 971199).

### 2.2. Procedures

Each subject underwent 8 treatments on separate days, with each subject performing up to 3 tests per week separated by at least one day. On each test day, subjects came to the facility (20 Victoria Street, 3rd floor, Toronto, ON, Canada) in the morning after a 10–14 h overnight fast. After being weighed, subjects gave 2 fasting blood samples obtained by finger-prick ~5 min apart. After the first fasting blood sample, subjects consumed a preload consisting of 200 mL water either alone or mixed (by hand, avoiding clumps) with one of 6 OBG sources (described below) each of which contained 4 g OBG of varying MW (Table 1). Subjects consumed water alone (Control) on 2 separate occasions and each of the OBG treatments once in randomized order. After the preload, a second fasting blood sample was obtained. Subjects then consumed a portion of white bread containing 51.5 g avCHO within 12 min. Subjects could choose to have, with each test meal, a drink of coffee, tea, or water, to which 30 mL 2% milk and/or artificial sweetener could be added; the drink chosen remained the same for all 8 treatments. After consuming the test meal, subjects rated the palatability of the test meal using a visual analogue scale (VAS) consisting of a 100 mm line anchored at the left end by “very unpalatable” and at the right end by “very palatable”. Subjects make a vertical mark along the line to indicate their perceived palatability. The distance from the left end of the line to the mark made by the subject was the palatability rating; the higher the value, the higher the perceived palatability. Further blood samples were obtained at 15, 30, 45, 60, 90, and 120 min after starting to eat. Subjects remained seated quietly during the 2 h of the test. After the last blood sample had been obtained, subjects were offered a snack and then permitted to leave.

Blood samples (2–3 drops each) were collected into 5 mL tubes containing sodium fluoride/potassium oxalate, mixed by rotating the tube vigorously, placed into a refrigerator until the end of the test session, then stored at −20 °C prior to glucose analysis using a YSI (Yellow Springs Instruments, Yellow Springs, OH, USA) analyser within 5 days.

### 2.3. Preparation of Test Meals and OBG Treatments

White bread was baked in a bread maker in loaves containing 500 g avCHO. The ingredients for each loaf (520 mL warm water, 708 g all-purpose flour, 14 g sugar, 8 g salt, and 13 g yeast) were placed into an automatic bread maker according to instructions, and the machine turned on. After the loaf had been made, it was allowed to cool for an hour, and then weighed and after discarding the crust ends, the remainder was divided into portions each of which weighed ~113 g and contained 9 g protein, 0.7 g fat, 54.4 g total carbohydrate, and 2.9 g dietary fiber. These portions were frozen prior to use, and reheated in the microwave prior to consumption.

The sources of OBG in 2 of the treatments were commercial oat products (OatWell^®^, DSM Nutritional Products, Heerlen, The Netherlands, termed OP1; and PromOat^®^, Tate & Lyle, London, UK, termed OP2), while the source of OBG in 4 of the treatments (MW1, MW2, MW3, and MW4) consisted of OP1 which had been treated to reduce the MW of the OBG it contained using a method based on Zurbriggen et al. [14] and Aktas-Akyildiz et al. [15]. The details of this procedure are described in Supplemental Information.

### 2.4. Measurement of OBG Viscosity and MW

The amount of OBG in the test sources was measured using the American Organization of Analytical Chemists approved method 995.16 [16]. The viscosity of the test OBG sources was measured using a method adapted from the American Association of Cereal Chemists International approved method 32-24.01 [17]. Viscosity measured by this method has previously been shown to correlate with post-prandial blood glucose response [18]. The MW of β-glucan was determined using a Gilson HPLC with a Shimadzu RF-551 fluorescence detector (excitation: 415 nm; emission: 450 nm) using a method adapted from previous research [19]. Details of these methods are described in Supplemental Information.

The characteristics of the OBG preloads are shown in Table 1. The MW and post-digestion viscosities of each OBG treatment were found to be significantly different from every other treatment.

### 2.5. Sample Size and Randomization

The primary endpoint of the study was glucose peak rise and the secondary endpoint glucose iAUC over 0–120 min (iAUC0-120). Based on our previous study [13], we estimated that 4 g of highly viscous OBG could reduce glucose peak rise by about 25%. We studied 16 subjects because, using the t-distribution and assuming an average coefficient of variation (CV) of within-individual variation of 20%, *n* = 16 subjects has a 90% power to detect a 25% difference in glucose peak rise with two-tailed *p* < 0.05. The order of treatments was randomized using a balanced Latin-square design [20]. The 16 orders created were assigned to subjects in the order they attended for the first visit after being recruited.

### 2.6. Data Management and Statistical Analysis

Data were entered into a spreadsheet by 2 different individuals and the values were compared to assure accurate transcription. Values for the glucose were missing for 4 (0.4%) of the 1024 blood samples due to clotted blood; 2 values at –5 min were replaced by the value at 0 min; 2 values at 15 min were imputed using a procedure described by Snedecor and Cochran [21]. The mean ± SD glucose concentration in the 113 fasting (0 min) samples in which duplicates could be measured was 4.37 ± 0.0409 mmol/L (analytical coefficient of variation (CV = 100 × SD/mean) of 0.9%). Glucose peak rise was the maximum glucose concentration minus the fasting concentration. Incremental areas under the curve (iAUC) were calculated using the trapezoid rule, ignoring area beneath the baseline [22], for the time interval of 0–120 min (iAUC0-120; secondary endpoint) and the time intervals of 0–45 min (iAUC0-45) and 45–120 min (iAUC45-120; additional endpoints). For calculation of iAUC and peak rise, fasting glucose was taken to be the mean of the first measurement of glucose in the 2 fasting samples. The time taken to reach the peak (time to peak) was taken to be the time of the maximum measured glucose concentration. The iAUC and peak rises for each OBG treatment were expressed relative to the mean iAUC or peak rise after the 2 tests of White Bread alone taken by the same subject. Relative response values >2 × SD greater than the mean were deemed outliers and replaced by the mean for the respective treatment.

For statistical analysis, the mean of the values for the 2 Control tests were considered as one treatment. Blood glucose concentrations and increments were subjected to a repeated-measures analysis of variance using the general linear model procedure (ANOVA) and examining for the main effects of time and treatment and the time × treatment interaction. After the demonstration of a significant time × treatment interaction, the difference between each OBG treatment and Control at each time was determined using Dunnett’s test. Values for iAUC and peak rise were analysed using ANOVA, examining for the main effect of the treatment. After the demonstration of a significant main effect, the significance of the difference between each OBG treatment and the Control was determined using Dunnett’s test. Differences were considered to be statistically significant if two-tailed *p* < 0.05. The significance of the regressions of log MW, log initial viscosity and log post-digestion viscosity of the 6 OBG treatments on the various outcomes were determined by ANOVA, with *p* < 0.05 taken to be the criterion for statistical significance.

## 3. Results

The test-meals were all consumed within the specified time and there were no adverse events or protocol deviations. All the OBG treatments were rated as being significantly less palatable than the Control (Table 2). Mean palatability was not related to MW (*p* = 0.51) or viscosity (*p* = 0.52) (not shown). Mean fasting glucose was similar for the 7 treatments (Table 2).

Compared to Caucasians, non-Caucasian subjects had higher iAUC45-120 (*p* < 0.05) and higher iAUC0-120 (*p* = 0.06), and subjects with BMI <25 kg/m^2^ had higher iAUC0-45, iAUC45-120 and iAUC0-120 than those with BMI ≥25 kg/m^2^. However, when expressed as a percentage of the Control, all subjects responded similarly regardless of their sex (not shown), age, ethnicity or BMI (Supplementary Table S2).

**Figure 1:**
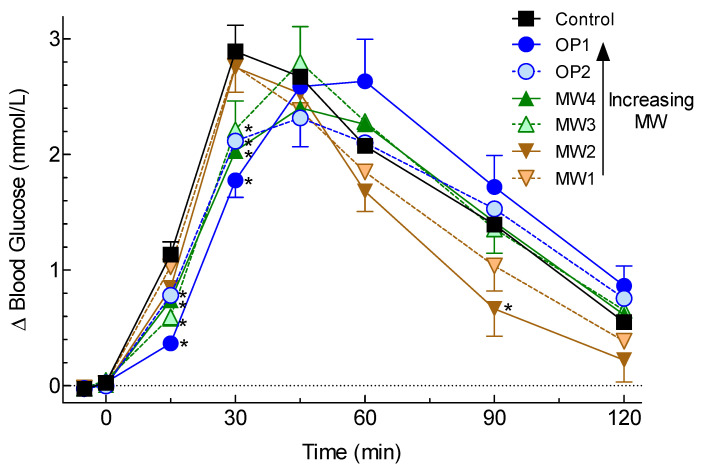
Glycemic responses elicited by the test meals. Values are means ± SEM for *n* = 16 subjects. The Control (200 mL water) and oat β-glucan preloads (MW1, MW2, MW3, MW4, OP1, and OP2 added to 200 mL water) were taken 2–3 min before consuming ~113 g white bread. * Significantly different from the Control by Dunnett’s test for two-tailed *p* < 0.05.

There were significant time×treatment interactions for blood glucose concentrations (*p* < 0.0001) and increments (*p* < 0.0001). The mean blood glucose increments after MW3, MW4, OP1, and OP2 were significantly less than after the Control at both 15 and 30 min, while increments after MW2 were significantly less than the Control at 90 min (Figure 1).

There was no significant difference among treatments for the primary endpoint of the glucose peak rise and no significant difference for the secondary endpoint of glucose iAUC0-120.

Additional analysis showed that the iAUC0-45 after MW3, MW4, OP1, and OP2 were significantly less than the Control, and, after MW4 and OP1, it took significantly longer for blood glucose to reach a peak than after the Control (Table 2). Glucose iAUC45-120 differed significantly among treatments, but none of the treatments differed from the Control (Table 2).

**Figure 2:**
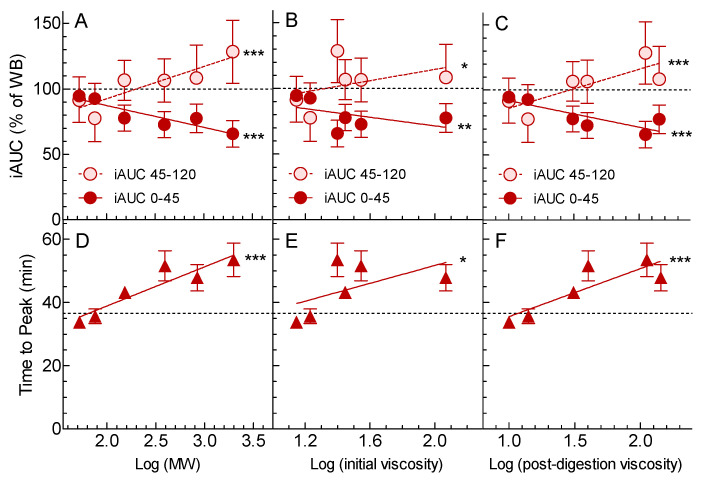
Relationships between glycemic response and β-glucan MW and viscosity. Values are means ± SEM for *n* = 16 subjects. Panels A, B, and C show incremental areas under the curve (iAUC) expressed as a percentage of that elicited by the Control; dark red circles are iAUC over 0–45 min, light red circles are iAUC over 60–120 min. Panels D, E, and F show the time taken to reach peak glucose in minutes. Panels A and D show the relationships between glycemic responses and log (MW); panels B and E between glycemic responses and log (initial viscosity); and panels C and F between glycemic responses and log (post-digestion viscosity). The black dashed lines show the value for the Control, and dark red solid and light red dashed lines are regression lines. Significance of the regressions by ANOVA: * *p* < 0.05; ** *p* < 0.01; *** *p* < 0.001.

When expressed as a % of Control, iAUC0-45 was significantly associated with log MW (*p* < 0.0001), log initial viscosity (*p* < 0.01), and log post-digestion viscosity (*p* = 0.006) (Figure 2). Ten-fold (1 log unit) increases in MW, initial viscosity, and post-digestion viscosity, respectively, were associated with ~17, ~16 and ~20% reductions in iAUC0-45 (Figure 2).

When expressed as a % of Control, mean iAUC45-120 was significantly associated with log MW (*p* = 0.002), log initial viscosity (*p* = 0.046), and log post-digestion viscosity (*p* = 0.0004).

The time it took blood glucose to reach the peak was significantly associated with log MW (*p* < 0.0001), log initial viscosity (*p* = 0.003), and log post-digestion viscosity (*p* < 0.0001) (Figure 2). Ten-fold (1 log unit) increases in MW, initial viscosity, and post-digestion viscosity, respectively, were associated with ~12, ~14, and ~15 min delays in the time to reach peak blood glucose (Figure 2).

## 4. Discussion

The results showed that there was no significant difference among treatments for the primary endpoint of glucose peak rise or the secondary endpoint of iAUC0-120 (Table 2). Thus, our a-priori hypothesis that the extent to which an OBG preload reduces the glycemic response (as measured by glucose peak rise) depends on its viscosity was not supported. However, post-hoc additional analysis showed significant differences among treatments for glucose iAUC0-45 and for the time taken to reach the peak (Table 2), with greater log MW and log viscosity being associated with lower iAUC0-45 min (*p* < 0.001) and a longer time to reach peak glucose (*p* < 0.001) (Figure 2).

**Figure 3:**
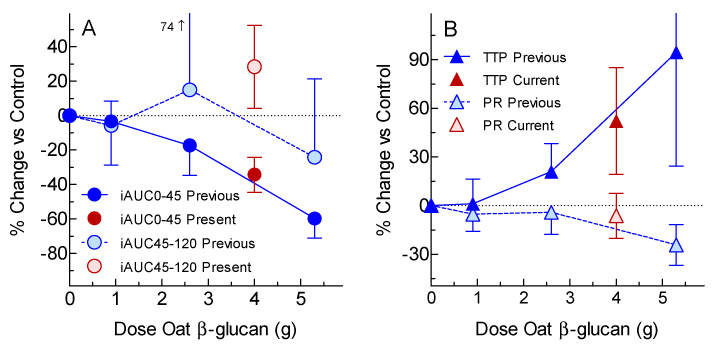
Current results compared to previous results for the same oat-bran treatment. Values are means ± 95% confidence intervals of the percentage change from control (0 g oat β-glucan). The previous study [13] tested the effects of a commercially available oat bran containing 0.9, 2.6 and 5.3 g of oat-β-glucan given as a preload on the glycemic response elicited by white bread in *n* = 10 subjects (blue symbols) using the same protocol as the present study. The present results for the same source of oat β-glucan used in the previous study (i.e., OP1) are shown with red symbols. Panel A shows the incremental area under the glucose curve (iAUC) from 0–45 min (dark circles) and from 45–120 min (light circles). Panel B shows the time to reach the peak (TTP; dark triangles) and peak rise (PR; light triangles).

The lack of any significant reduction in iAUC0-120 and peak rise after 4 g OBG preloads was surprising, given our previous demonstration that, when 0.9, 2.6 and 5.3 g doses of OBG were given as a preload before consuming a 50 g avCHO portion of white bread, OBG reduced both glucose peak rise and iAUC0-120 in a dose-dependent fashion [13]. However, since only the 5.3 g OBG dose elicited statistically significant reductions, it is possible that that the 4 g dose used here may not have been large enough to elicit significant reductions in peak rise of iAUC0-120. Furthermore, we did not report results for iAUC0-45, iAUC45-120, and time to peak in the previous publication [13]. One of the sources of OBG included in the present study, OP1, was also used as the source of OBG in our previous study [13]. When the results for 4 g OBG from OP1 from the present study are plotted on dose-response curves for iAUC0-45, iAUC45-120, time to peak, and peak rise from the previous study [13], the results from the present study are consistent with those from the previous study, since the 95% confidence intervals (95% CI) from the 2 studies overlap (Figure 3). Taken together, the results of these 2 studies suggest that, when high MW OBG is taken as a preload, increasing the dose of OBG without changing MW reduces glucose peak rise, iAUC0-45, and iAUC0-120, and increases the time to peak in a dose-dependent fashion, whereas reducing the MW of 4 g of OBG only affects glucose iAUC0-45 and time to reach the peak. However, the effects of reducing the MW of preloads containing <4 g or >4 g OBG may differ from those seen here.

It is generally considered that the effect of OBG on glycemic responses is determined by its ability to increase the viscosity of the contents of the gastrointestinal tract. The viscosity of OBG can be decreased either by decreasing the MW of OBG or by decreasing the amount of OBG consumed. When OBG is mixed with the food consumed, the glucose peak rise is reduced in proportion to log (MW × C), where C is the concentration of OBG in the intestine [23,24]. Nevertheless, when OBG is mixed into a glucose solution, the effects of viscosity on glycemic response are not fully understood. For example, reducing the viscosity of an OBG-containing glucose solution by reducing MW significantly increased the glucose peak rise and iAUC0-120, but the same reduction in glucose solution viscosity obtained by increasing the volume of water in the OBG-containing glucose solution had no effect on glucose peak rise or iAUC0-120 [25]. This result is consistent with observations that high viscosity meals are diluted in the stomach by oral and gastric secretions more than low viscosity meals [26]. Furthermore, the viscosity of OBG in the small intestine may differ from the viscosity of test-meals because the digestion process may release OBG from the food matrix. This is illustrated in the present results where the post-digestion viscosities of OP1 and OP2 were higher than their initial viscosities; this may explain why post-digestion viscosity was more closely correlated to the glycemic outcomes than initial viscosity (Figure 2). However, the precise mechanisms by which OBG reduces glycemic responses are not fully understood.

One weakness of this study is that the effect of altering MW using different doses of OBG was not determined, and thus our conclusions only apply to a dose of 4 g. Also, the study was powered to detect a 25% reduction in peak rise, but was underpowered to detect the 18% reduction observed for OP2 vs. the control. Post-hoc power analysis indicates that *n* = 20 subjects would have provided 80% power to detect the 18% difference, and *n* = 25 subjects would have provided 90% power. Finally, we did not measure serum insulin responses. When OBG is mixed with the test meal consumed, the insulinemic response is reduced in proportion to the reduction in glucose [27,28]. Here, however, as the MW of OBG in a preload increased, we found a delay in the rise and fall of postprandial glucose but no reduction in peak rise. We would expect the delayed rise in glucose to be accompanied by a delayed rise in insulin, but we do not know if the lack of effect on the glucose peak rise and delayed fall in glucose were associated with lower or higher insulin responses. Such information would be useful to better understand the mechanism of this effect.

Our results raise the question of whether delaying the rise in postprandial glucose (shown here by the reduced glucose iAUC0-45 and delayed time to peak), in the absence of a reduction in peak-rise or iAUC0-120, is beneficial for the prevention or treatment of diabetes. There is evidence that blood glucose 30 or 60 min after a 75 g oral glucose tolerance test is a similar or better predictor of future type 2 diabetes (T2D) than the traditional 120 min [29]. This suggests that a delayed rise in postprandial blood glucose may be associated with reduced risk for T2D. Consistent with this are the results of a study showing that when subjects with T2D took 5 g of guar granules prior to consuming mashed potatoes (23 g avCHO), the glucose peak rise was delayed by 30–45 min, but there was no significant effect on glucose peak rise or iAUC; however, 5 g of guar mixed into the mashed potato test-meal reduced glucose iAUC ~40% [12]. Nevertheless, 4 g of guar granules twice daily before meals for 6 wk reduced HbA1c to the same extent as 8 g of guar per day incorporated into bread, with both guar treatments eliciting a significantly greater reduction in HbA1c than the control treatment [30]. Thus, OBG preloads which delay the rise in blood glucose without reducing it may be beneficial in T2D.

A 10–15 min delay in the postprandial glucose rise may also be useful in people with type 1 diabetes using subcutaneously administered insulin. Modern rapid-acting insulins begin to act 5–15 min after insulin administration [31]. Delaying meal consumption for 15 min after rapid-acting insulin administration reduced the glycemic response compared to eating the same meal immediately before or after insulin administration [32,33]. Thus, the ability of an OBG preload to delay the rise in blood glucose after eating could be useful in the management of type 1 diabetes and may warrant investigation. In addition, long-term OBG consumption may have beneficial effects on glycemic control by virtue of its fermentation in the large intestine with the production of short-chain fatty acids [34] and potential alteration of the colonic microbiota [35].

The effects of the OBG treatments on glucose iAUC0-45 were nearly opposite to those on iAUC45-120 (Figure 2). This likely reflects the different kinetics of glucose absorption of the treatments along the upper GI tract depending on MW and viscosity. The Control, MW1, and MW2, with lower MWs (≤76 kDa) and viscosities (≤14 cP) than MW3, MW4, OP1, and OP2, may have emptied from the stomach more quickly and been absorbed from the small intestine more rapidly, resulting in a high early glucose response (iAUC0-45) and a low late glucose response (iAUC45-120). In contrast, glucose absorption from MW3, MW4, OP1, and OP2, with higher MWs (153–1980 kDa) and viscosities (31–143 cP), may have been delayed, resulting in a lower early glucose response, a delayed peak, and higher late glucose response. The inverse correlations of iAUC0-45 and iAUC45-120, respectively, explain the lack of effect of the OBG treatments on iAUC0-120.

The pre-determined primary endpoint for this study was glucose peak rise. We previously showed that incorporating 4 g OBG with MW 132 kDa into a muffin containing 48 g avCHO had no significant effect on glucose peak rise, whereas 4 g of native OBG (MW ~2200 kDa) reduced glucose peak rise by 40% [23]. Although a similar range of MWs was investigated here (52 to 1980 kDa), altering the MW of 4 g OBG ingested separately from the avCHO had no significant effect on glucose peak rise, possibly because the viscous preloads were quickly diluted by oral and gastric secretions as the bread was being eaten. However, we used a preload containing 4 g OBG with a test meal of white bread containing ~50 g avCHO. The results might differ with different doses of OBG or with different types or sizes of the test meal.

## 5. Conclusions

We conclude that, when 4 g OBG is taken as a preload, reducing MW does not affect the peak rise or iAUC0-120, but rather accelerates the rise in blood glucose and reduces the time it takes for blood glucose to reach the peak. However, this conclusion is based on additional post-hoc comparisons of iAUC0-45 and the time to peak, and need to be confirmed in a subsequent study.

## Figures and Tables

**Table 1:** Composition of the Control oat β-glucan preloads.

Preload	Amount (g)	Protein (g)	Fat (g)	Total Carb. (g)	Total Dietary Fiber (g)	Oat β-Glucan			
						Amount (g)	MW (kDa)	Viscosity (cP)	
								Initial	PD *
**Control**	**0**	**0**	**0**	**0**	**0**	**0**	-	-	-
MW1	7.48	na	na	na	5.0	4.0	52 ± 5	14 ± 4	10 ± 1
MW2	7.41	na	na	na	5.1	4.0	76 ± 8	17 ± 3	14 ± 1
MW3	8.28	na	na	na	5.7	4.0	153 ± 5	28 ± 2	31 ± 1
MW4	10.42	na	na	na	5.1	4.0	393 ± 31	35 ± 5	40 ± 1
OP1	13.7	3.2	0.7	8.4	7.2	4.0	1980 ± 265	25 ± 1	112 ± 9
OP2	11.90	0.4	0.1	10.7	5.5	4.0	841 ± 110	117 ± 7	143 ± 7

The Control preload was 200 mL water; the oat β-glucan preloads (MW1, MW2, MW3, MW4, OP1, and OP2) were added to 200 mL water. Values for protein, fat and total carbohydrate (Carb.) were provided by the sponsors (“na” = not available). Total dietary fiber was measured by Eurofins CLF Specialised Nutrition Testing Services GmBH, Professor-Wagner-Straße 11, D-61381 Friedrichsdorf, Germany. Values for MW and viscosity are means ± SD of quadruplicate measures. * PD = post-digestion.

**Table 2:** Palatability, fasting glucose, and measures of glycemic response.

Preload	Palatability (mm)	Fasting Glucose (mmol/L)	Peak Rise (mmol/L)	Incremental Area under the Curve (mmol × min/L)			Time to Peak (min)
				0–45 min	45–120 min	0–120 min	
Control	68 ± 6	4.39 ± 0.08	3.10 ± 0.27	80 ± 6	118 ± 15	198 ± 20	37 ± 3
MW1	47 ± 7 *	4.48 ± 0.07	2.89 ± 0.23	75 ± 6	98 ± 11	173 ± 15	34 ± 2
MW2	46 ± 6 *	4.38 ± 0.08	2.89 ± 0.23	73 ± 6	85 ± 11	159 ± 14	36 ± 2
MW3	34 ± 7 *	4.34 ± 0.06	2.93 ± 0.30	63 ± 6 *	123 ± 16	186 ± 21	43 ± 2
MW4	34 ± 8 *	4.44 ± 0.08	2.70 ± 0.32	60 ± 7 *	122 ± 17	181 ± 22	52 ± 5 *
OP1	43 ± 7 *	4.47 ± 0.08	2.90 ± 0.31	52 ± 4 *	143 ± 18	195 ± 21	53 ± 5 *
OP2	45 ± 7 *	4.40 ± 0.07	2.55 ± 0.29	61 ± 5 *	122 ± 18	183 ± 21	48 ± 4
*p* **	<0.0001	0.095	0.19	<0.0001	0.002	0.20	<0.0001

Values are means ± SEM for *n* = 16 subjects. The Control preload was 200 mL water; the oat β-glucan preloads (MW1, MW2, MW3, MW4, OP1 and OP2) were added to 200 mL water. Preloads were taken 2–3 min before consuming ~113 g white bread. * Significantly different from the Control by Dunnett’s test for two-tailed *p* < 0.05. ** Significance of main effect of treatment by ANOVA.

## Supplementary Materials

The following are available online at https://www.mdpi.com/2072-6643/12/8/2275/s1, Table S1: Demographic details of Caucasian and non-Caucasian participants, Table S2: Glycemic responses in Caucasian and non-Caucasian participants.
