# Supplementary material for: Effect of Varying Molecular Weight of Oat β-Glucan Taken just before Eating on Postprandial Glycemic Response in Healthy Humans
Source: Nutrients. 2020 Jul 29;12(8):2275. doi: 10.3390/nu12082275 (PMC7469033; doi:10.3390/nu12082275)
Supplement: Supplementary file 1 [file nutrients-12-02275-s001.pdf]

Hydrolysis of Oat β-glucan (OBG)

OP1 was pre-conditioned by adding water to reach 30% moisture content. Enzymatic hydrolysis was performed with Depol 740L (Biocatalyst Ltd., UK) which contained 9888 nkat/ml of β-glucanase activity as measured with 1% barley β-glucan as substrate in 100mM phosphate buffer at pH 6 and 50 °C. Different enzyme dilutions were tested in order to find the correct enzyme dosages for the production of samples with target MW. The pre-conditioned OP1 was fed to the extruder together with determined enzyme dilutions. The dough-like mass resulting from the extrusion was incubated for 1 h at 50°C. The sample was inactivated and extracted by adding boiling water and homogenized. The homogenized material was centrifuged and the liquid fraction containing water-soluble compounds was separated, quickly frozen and freeze-dried. The freeze-dried samples were ground using a Retsch SM300 cutting mill.

Measurement of OBG viscosity and molecular weight (MW)

OBG viscosity was measured as follows: slurries of the OBG treatments were prepared in the same solid to liquid ratio as were consumed in vivo testing; 1 part solid to 50 parts liquid. Twenty-five (25) mL of buffer (20mM H2NaPO4 +10 mM NaCl; pH 6.9 with 0.02% NaN3) was weighed into an RVA canister and 0.5 g of sample was mixed into the buffer avoiding the creation of lumps. The canister was placed on the RVA (model 4500, Perten, North Ryde, Australia) and mixed at 37°C and 160 rpm for 5 min. The viscosity at the end of 5 min was recorded and designated the initial viscosity. Enzymes, 3.5 U α-amylase (Bacillus licheniformis, 3,000 U/mL, E-BLAAM, Megazyme, Bray, Ireland) and 30 U protease (Bacillus licheniformis, 300 U/mL, E-BSPRT, Megazyme, Bray, Ireland) were added to the slurry. The canister was returned to the RVA and the slurry was mixed at 37°C and 160 rpm for 120 min. The final viscosity measurement was recorded. In this method, the viscosity is measured both before (initial) and after (post-digestion) treatment with α-amylase and protease for 2 h at 37°C to remove the contribution of starch and protein to viscosity and approximate the relative differences in viscosity expected as the meal bolus moves through the upper intestine.

The MW of OBG was measured as follows: approximately 40 mg of each OBG source was dispersed in 5 mL of buffer, heated in a boiling water bath for 1 h with constant mixing, cooled to room temperature and centrifuged for 30 min at 5000×g. The supernatant was decanted, diluted as required with 0.05% NaN3 and filtered through a 0.80 μm filter. Solutions were injected into two Shodex (Showa Denko K.K., Tokyo, Japan) OHpak SB-806 M columns in series (with OHpak guard) in 0.1M Tris buffer, pH 8.0, at 1 mL/min. To detect the β-glucan, post-column addition of 7.5 mg/L calcofluor (Fluorescent brightener 28, Sigma-Aldrich, St. Louis, MO) in 0.1M Tris buffer was used. Each source of OBG was tested in quadruplicate. Commercial β-glucan standards (Megazyme International, Bray, County Wicklow, Ireland) were used to construct a standard curve of log(peak molecular weight) vs. retention time and weight average molecular weights (Mw) were calculated.

Table 1. Demographic details of Caucasian and non-Caucasian participants.

**Ethnicity**	**Sex (M:F)**	**Age (y)**	**Height (cm)**	**Weight (kg)**	**BMI (kg/m²)**
Caucasian	4:3	45.7±8.6	172±10	81.8±8.0	27.8±2.1
non-Caucasian*	5:4	36.4±10.8	168±7.2	67.4±8.1	23.9±1.6
p	0.95	0.084	0.36	0.003	0.001

Values are means±SD. * Non-Caucasian included: 4 East- or South-East Asian, 2 South Asian, 2 Hispanic and 1 mixed Aboriginal/Caucasian.

Table 2. Glycemic responses in Caucasian and non-Caucasian participants.

**Glucose Measure**	Units	Ethnicity		Age (years)		BMI (kg/m²)	
		Cauc (n = 7)	non-Cauc (n = 9)	<40 (n = 8)	≥40 (n = 8)	<25 (n = 7)	≥25 (n = 9)
**Fasting**	mmol/L	4.52 ± 0.36	4.33 ± 0.13	4.36±0.27	4.46±0.13	4.36±0.27	4.45±0.14
Peak Rise	mmol/L	2.60 ± 0.73	3.05 ± 1.15	2.66±1.00	3.05±0.50	3.32±0.92	2.49±0.47
iAUC 0-45 min	mmol×min/L	60 ± 19	71 ± 20	65 ± 20	68 ± 10	77 ± 17	58 ± 9 [2]
	% of Control	75 ± 8	84 ± 3	82 ± 16	78 ± 8	85 ± 15	77 ± 8
iAUC 60-120 min	mmol×min/L	88 ± 28	138 ± 52 [2]	120 ± 51	112 ± 25	151 ± 39	89 ± 20 [3]
	% of Control	108 ± 5	100 ± 9	105 ± 22	101 ± 11	106 ± 22	101 ± 11
iAUC 0-120 min	mmol×min/L	148 ± 42	209 ± 69 [1]	185 ± 67	179 ± 34	228 ± 51	146 ± 26 [3]
	% of Control	91 ± 14	93 ± 18	95 ± 16	89 ± 8	97 ± 16	88 ± 8
Peak Time	min	41.8 ± 6.4	44.2 ± 7.6	43.5 ± 7.2	42.7 ± 3.6	45.9 ± 7.1	41.0 ± 6.5

Values are means ± SD. Cauc = Caucasian; non-Cauc = non-Caucasian. Test-meals consisted of a Control (water) or oat β-glucan (4g OBG mixed into water) preload followed by ~113g white bread. Values for mmol/L, mmol×min/L or min are means of all treatments (water alone and the 6 OBG preloads); values for % of Control (water alone) are the means of the 6 OBG treatments each expressed as a % of that for Control (white bread alone). None of the differences between groups is significant ($p > 0.1$) except for those with superscripts. [1,2,3] Significance of difference from Cauc or BMI<25; 1 = $p = 0.06$, 2 = $p < 0.05$, 3 = $p < 0.01$.
